# Factors affecting the long-term outcomes of idiopathic membranous nephropathy

**DOI:** 10.1186/s12882-017-0525-6

**Published:** 2017-03-27

**Authors:** Hyuk Huh, Hajeong Lee, Jung Pyo Lee, Dong Ki Kim, Sohee Oh, Yun Kyu Oh, Yon Su Kim, Chun Soo Lim

**Affiliations:** 1 Department of Internal Medicine, Seoul National University Hospital, Seoul, Republic of Korea; 2 Department of Internal Medicine, Seoul National University Boramae Medical Center, 20 Boramae-ro 5-gil, Dongjak-gu, Seoul, 07061 Republic of Korea; 3 Department of Internal Medicine, Seoul National University College of Medicine, Seoul, Republic of Korea; 4 Department of Biostatistics, Seoul National University Boramae Medical Center, Seoul, Republic of Korea

**Keywords:** Idiopathic membranous nephropathy, Nephrotic syndrome, Prognosis, Proteinuria, Renal survival

## Abstract

**Background:**

We attempted to describe the clinical features and determine the factors associated with renal survival in idiopathic membranous nephropathy (iMN) patients with nephrotic syndrome (NS) and to determine the factors associated with spontaneous complete remission (sCR) and progression to NS in iMN patients with subnephrotic proteinuria.

**Methods:**

This retrospective study involved 166 iMN patients with NS and 65 patients with subnephrotic proteinuria. The primary end point was a doubling of serum creatinine or initiation of dialysis. In patients with subnephrotic proteinuria, we determined the factors associated with sCR and factors associated with progression to NS.

**Results:**

Remission of NS was achieved in 125 out of 166 patients (75.3%). Of those who reached remission, 26 patients (20.8%) experienced relapse that was followed by second remission. The relapse or persistence of proteinuria was associated with the primary end points (hazard ratio [HR] = 12.40, *P* = 0.037, HR = 173, *P* < 0.001, respectively). In patients with subnephrotic proteinuria, sCR occurred in 35.4% of the patients. The patients with sCR had lower proteinuria and serum creatinine levels and higher serum albumin concentrations at baseline. The serum albumin level at diagnosis was a prognostic factor for progression to NS (Odds ratio [OR] = 0.015, *P* < 0.001).

**Conclusions:**

The occurrence of relapse or persistence of proteinuria had negative effects on renal survival in iMN patients with NS, and low serum albumin levels at baseline were associated with non-achievement of sCR and progression to NS.

## Background

Idiopathic membranous nephropathy (iMN) is the most common cause of adult onset nephrotic syndrome (NS) [1, 2]. Clinical presentations of iMN vary from subnephrotic-range proteinuria regarded as asymptomatic urinary abnormality to NS with heavy proteinuria. From the studies on the natural history of iMN, it was suggested that many untreated patients had stable renal function and possibility of spontaneous remission [3]. Approximately one-third of the patients experience spontaneous remission, another third show persistent proteinuria, and the remaining third progress to end-stage renal disease (ESRD). Approximately 20–30% of untreated iMN patients progress to ESRD [4]. These variable courses of iMN lead to great difficulties when physicians need to decide the treatment regimen.

Many researchers have attempted to identify the risk factors for poor prognosis. Several studies showed that male sex, old age (>50 years old), hypertension, massive proteinuria (>10 g/24 h), and elevated serum creatinine concentration at the time of renal biopsy are poor prognostic factors of iMN [5–8]. Also, Polanco et al. reported that spontaneous remission was predicted based on baseline serum creatinine levels and proteinuria extent, treatment with angiotensin-converting enzyme inhibitors or angiotensin-receptor antagonists, and a >50% decline in proteinuria from baseline during the first year of follow-up [9]. Among these factors, persistent heavy proteinuria was the most reliable predictor of life-threatening complications and poor renal outcomes in iMN patients. On the basis of these results, aggressive treatments have been introduced to induce complete or partial remission or to reduce the amount of proteinuria in patients with poor prognostic factors.

Not only the aforementioned demographic and laboratory parameters at diagnosis but also response to treatment could be major predictors of renal survival. In patients with NS, whether remission is achieved or not could affect long-term outcomes [10]. The patients who had subnephrotic-range proteinuria during the observation period had better outcomes than patients who progressed from subnephrotic-range proteinuria to nephrotic-range proteinuria [11].

Despite we consider the proteinuria levels as an important prognostic factor, it is still uncertain whether relapse or persistence of proteinuria has a negative impact on long-term renal outcomes in iMN patients. Therefore, we performed a retrospective study to describe the clinical features and determine the factors associated with renal survival in iMN patients with NS and to determine the factors associated with spontaneous complete remission (sCR) and progression to NS in iMN patients with subnephrotic-range proteinuria. Besides determining the prognostic factors, we also expected to gain additional information regarding differences according to geographical regions or populations.

## Methods

### Study population

We attempted to recruit biopsy-proven adult iMN patients by reviewing medical records from the archives of the Department of Pathology at the Seoul National University Hospital. Patients with systemic diseases such as rheumatic diseases, malignant tumours, or infections with hepatitis B or C virus, and other diseases associated with secondary membranous nephropathy (MN) were excluded. From January 1989 to December 2012, a total of 231 patients were diagnosed with idiopathic or primary MN through renal biopsy. We classified the patients into 2 groups according to the level of proteinuria at the time of renal biopsy. Of them, 65 patients (28.1% of total patients) presented with subnephrotic range proteinuria, and 166 patients (71.9%) had NS that was defined as proteinuria of 3.5 g/day (or urine protein:creatinine ratio [uPCR] 3.5 g/g) or more and the presence of hypoalbuminemia.

### Study design

Information regarding the demographic, clinical, and laboratory variables was collected via medical record review. We determined the factors affecting the long-term outcomes separately according to the groups. First, concerning patients with NS, we analysed the factors affecting the renal survival including the impact of relapse or persistence of proteinuria. Second, in patients with subnephrotic-range proteinuria, we evaluated the factors predicting spontaneous remission of proteinuria or progression to NS. The primary outcome was the doubling of serum creatinine concentration or development of ESRD in iMN patients with NS or subnephrotic proteinuria. The secondary outcome was sCR or progression to NS in iMN patients with subnephrotic-range proteinuria.

### Definition

We adopted the definition of the KDIGO practice guideline on glomerulonephritis, presented in 2012 [12]. Complete remission (CR) was defined as urinary protein excretion <0.3 g/day (uPCR < 0.3 g/g) confirmed by 2 values at least 1 week apart, accompanied by normal serum albumin concentration and normal serum creatinine level. Partial remission (PR) was defined as urinary protein excretion <3.5 g/day (uPCR < 3.5 g/g) and ≥50% reduction from the peak values confirmed by 2 values at least 1 week apart, accompanied by an improvement or normalisation of the serum albumin concentration and stable serum creatinine level. In addition, we defined proteinuria less than 0.3 g/day (uPCR < 3.5 g/g) as subnephrotic-range proteinuria and a case that meets aforementioned conditions of remission without using immunosuppressive agents as sCR. The patients who showed spontaneous PR or reached remission through immunosuppressive therapy were not considered as having sCR. After remission was achieved, urinary protein excretion >0.3 g/day (uPCR > 0.3 g/g) in CR or ≥50% increase in the lowest values in patients with PR confirmed by 2 values at least 1 week apart with or without decline of serum albumin concentration and/or rise of serum creatinine was defined as relapse. The progression to NS was defined as development of NS in patients who had subnephrotic-range proteinuria at the time of diagnosis.

### Statistical analysis

Numerical variables were expressed as the mean ± standard deviation (SD), and non-parametric variables were expressed as median and range. For group comparisons, 2-sample *t*-test, Mann-Whitney *U* test, analysis of variance (ANOVA), and Kruskal-Wallis test were applied. Categorical variables were expressed as numbers and percentages and compared using the *χ* ^2^ test or Fisher’s exact test. Renal event-free survival rate was calculated by the Kaplan-Meier method. The difference in survival rates between groups was examined by the log-rank test. The relationship of the covariates to renal survival was evaluated using the Cox proportional hazards model, yielding the hazard ratio (HR) and 95% confidence interval. Additionally, to assess the effects of variables on sCR or progression to NS in subnephrotic-range proteinuria patients, univariate and multivariate logistic regression analyses were conducted, yielding unadjusted odds ratio (OR) and adjusted OR, respectively. Analyses were performed using the IBM SPSS statistics (version 21.0, Chicago, IL, USA). All tests were 2-tailed, with *P* < 0.05 considered statistically significant.

## Results

### Patients with NS

The baseline characteristics of the patients with NS are listed in Table 1. In all, 32 (19.4%) patients had an estimated glomerular filtration rate (eGFR) lower than 60 mL · min^−1^ · 1.73 m^−2^ at diagnosis. Seventy (42.2%) patients presented with heavy proteinuria (>8 g/g creatinine) and only 13 (7.8%) patients presented with urinary protein excretion <4 g/g creatinine at the time of renal biopsy. The median follow-up duration was 123 months (67–199). Renin-angiotensin-aldosterone system (RAAS) blockades, other anti-hypertensive agents (including beta-blockers and calcium channel blockers), and HMG-CoA reductase inhibitors (statins) were used for conservative management in 126 (75.9), 82 (49.4), and 123 (74.1%) patients, respectively. Conservative measures comprised RAAS blockades, and other non-immunosuppressive agents were applied to 30 (18.1%) patients. Although immunosuppressive regimens were not uniform according to the different therapeutic protocols, 136 (81.9%) patients were treated with immunosuppressive agents (Table 2).

**Table 1:** Baseline characteristics of patients

	Nephrotic syndrome (*N* = 166)	Subnephrotic proteinuria (*N* = 65)
Age (years)	53 ± 13	49 ± 12
BMI (kg/m^2^)	24.52 ± 2.90	23.65 ± 2.66
Male	103 (62%)	32 (49.8%)
SBP (mmHg)	130 ± 19	123 ± 14
DBP (mmHg)	82 ± 12	79 ± 8
Serum creatinine (mg/dl)	0.99 ± 0.39	1.03 ± 0.65
CKD stage according to eGFR		
Stage I	61 (37%)	28 (43.1%)
Stage II	72 (43.6%)	28 (43.1%)
Stage III – V	32 (19.4%)	9 (13.9%)
Serum albumin (g/dl)	2.41 ± 0.55	3.17 ± 0.70
Proteinuria (g/g creatinine)	8.18 (5.10–10.35)	2.11 (1.88–2.32)
> 8	70 (42.2%)	–
4–8	72 (43.4%)	–
< 4	13 (7.8%)	65

Data are presented as means ± SD, number (% of total) or median (range)

*SD* Standard deviation, *BMI* Body mass index, *SBP* Systolic blood pressure, *DBP* Diastolic blood pressure, *CKD* Chronic kidney disease, *eGFR* estimated glomerular filtration rate, estimated with Modification of Diet in Renal Disease equation

**Table 2:** Characteristics and outcomes according to the treatments in patients with nephrotic syndrome

	Conservative management (*N* = 30)	Oral corticosteroid (*N* = 22)	Oral corticosteroid + cyclophosphamide (*N* = 100)	Oral corticosteroid + cyclosporine (*N* = 14)
Age^a^ (years)	51	47	57	44
SBP (mmHg)	128	125	130	135
Proteinuria^a^(g/g creatinine)	6.43	6.95	8.93	8.53
Serum Albumin^a^(g/dL)	2.8	2.5	2.3	2.3
Serum creatinine (mg/dl)	0.91	0.93	1.03	0.92
Total cholesterol^a^(mg/dl)	277	354	354	321
Treatments				
RAAS blockers	26 (86.7%)	13 (59.1%)	75 (75%)	12 (85.7%)
Anti-HTN	13 (43.3%)	11 (50%)	51 (51%)	7 (50%)
Statin	22 (73.3%)	10 (45.5%)	79 (79%)	12 (85.7%)
Remission	26 (86.7%)	17 (77.2%)	73 (73%)	9 (64.3%)
Relapse	2/26 (7.7%)	3/17 (17.6%)	16/73 (21.9%)	5/9 (55.6%)
Persistence	4 (13.4%)	5 (22.8%)	27 (27%)	5 (35.7%)
End points^b^	2 (6.7%)	4 (18.2%)	13 (13%)	3 (21.4%)

Data are presented as mean, number (% of total)

^a^ *P* < 0.01, Difference between groups; *RAAS blockers* Renin-angiotensin-aldosterone system blockers, *Anti-HTN* Anti-hypertensive medication

^b^End points include doubling of serum creatinine concentration compared to baseline level and development of end stage renal disease

Remission of NS was achieved in 125 (75.3%) of 166 NS patients. Among the remission-induced patients, 75 (60.0%) patients maintained remission until the end of the follow-up period. Twenty-six (20.8%) patients experienced relapse of proteinuria: 2 in the conservative treatment group, 3 in the corticosteroids alone group, 16 in the corticosteroid plus cyclophosphamide group, and 5 in the corticosteroid plus cyclosporine group (Table 2). After the first relapse occurred, second-line immunosuppressive agents induced second remission in all the patients. Of the 30 patients that were managed conservatively, 26 (86.7%) patients achieved spontaneous remission. The remaining 4 patients did not achieve remission and had persistent proteinuria. Although the patients treated conservatively seemed to have had better prognosis, we should consider the disease severity at baseline. The conservatively treated patients had significantly lower proteinuria (*P* = 0.018) and higher serum albumin concentration (*P* < 0.001) than the groups treated with immunosuppressive agents (Table 2).

**Fig. 1:**
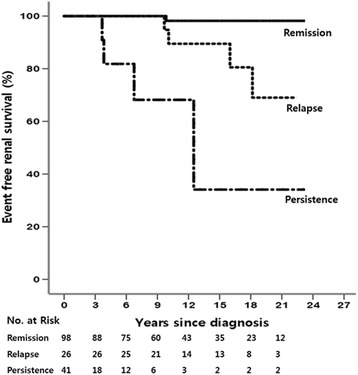
Kaplan-Meier curve of event-free renal survival according to the clinical courses in patients with nephrotic syndrome (*P* < 0.001)

The primary composite end-point, defined as the doubling of serum creatinine or development of ESRD, occurred in 22 (13.3%) patients. As shown in Table 3, on adjusting for age, gender, systolic blood pressure, serum creatinine, serum albumin, and proteinuria level, patients who experienced relapse showed poor outcomes (Hazard ratio [HR] = 12.40, *P* = 0.037). Furthermore, iMN patients who did not achieve remission had poorer long-term outcomes (HR = 173, *P* <0.001). The event-free renal survival rate was very good in patients with persistent remission. However, the survival rate was the poorest in patients who never reached remission. The patients who experienced relapse had intermediate long-term prognosis (Fig. 1).

**Table 3:** Multivariate Cox proportional hazards model for ESRD or doubling of serum creatinine in patients with nephrotic syndrome

	Hazard ratio	*P-* value
Relapse	12.40 (1.17–131.6)	0.037
Persistence	173 (11.2–2660)	0.001

Adjusted for age, gender, Systolic blood pressure, serum creatinine concentration, serum albumin concentration, serum cholesterol concentration, and quantity of urine protein

### Patients with subnephrotic range proteinuria

The baseline characteristics are listed in Table 1. Even in this group, 9 patients had decreased renal function (eGFR < 60 mL · min^−1^ · 1.73 m^−2^). The sCR was achieved in 23 (35.4%) patients. There were significant differences in serum albumin concentration, serum cholesterol level, and level of proteinuria between the sCR group and those who did not reach sCR (Table 4). Progression to NS occurred in 26 patients among 65 subnephrotic patients. Among sCR-achieved 23 patients, NS developed only in 2 patients, and among non-sCR-induced 42 patients, NS developed in 24 patients. On comparing patients with progression to NS and those with non-progression, the serum albumin levels were found to be low, and the serum cholesterol concentrations and proteinuria level, high (Table 5).

**Table 4:** Comparisons of clinical characteristics according to the spontaneous complete remission in patients with subnephrotic range proteinuria

	Spontaneous CR (*N* = 23)	No spontaneous CR^a^ (*N* = 42)	*P*-value
Male	12 (52.2%)	20 (47.6%)	0.463
Age (years)	46 ± 13	51 ± 11	0.065
BMI (kg/m^2^)	23.9 ± 3.2	23.5 ± 2.4	0.501
Hypertension	3 (13%)	13 (31%)	0.109
Serum creatinine (mg/dl)	0.90 ± 0.18	1.11 ± 0.80	0.230
Serum albumin (g/dl)	3.58 ± 0.52	2.95 ± 0.68	<0.001
Serum cholesterol (mg/dl)	217.7 ± 64.0	264.3 ± 92.1	0.043
CKD stage according to eGFR			0.103
Stage I	10 (43.5%)	18 (42.9%)	
Stage II	13 (56.5%)	15 (35.7%)	
Stage III	–	5 (11.9%)	
Stage IV-V	–	4 (9.6%)	
Serum hemoglobin (g/dL)	13.6 ± 1.6	12.6 ± 2.2	0.074
Proteinuria (g/g creatinine)	1.68 ± 0.88	2.36 ± 0.81	0.003

*BMI* Body mass index, *CKD* Chronic kidney disease, *eGFR* estimated glomerular filtration rate, estimated with Modification of Diet in Renal Disease

^a^Group of ‘No spontaneous CR’ include patients that had spontaneous partial remission or remission through immunosuppressant

Data are presented as number (% of total) or means ± SD

**Table 5:** Comparisons of clinical characteristics according to disease progression in patients with subnephrotic range proteinuria

	Progression to NS (*N* = 26)	No progression^a^ (*N* = 39)	*P*-value
Male	9 (34.6%)	23 (58.9%)	0.124
Age (years)	53 ± 11	47 ± 13	0.088
BMI (kg/m^2^)	23.5 ± 2.3	23.9 ± 2.8	0.513
Hypertension	8 (28.6%)	8 (20.5%)	0.558
Serum creatinine (mg/dl)	0.90 ± 0.18	1.11 ± 0.80	0.230
Serum albumin (g/dl)	2.67 ± 0.61	3.36 ± 0.48	<0.001
Serum cholesterol (mg/dl)	274.1 ± 92.4	225.3 ± 62.4	0.018
CKD stage according to eGFR			0.399
Stage I	14 (53.8%)	14 (35.9%)	
Stage II	10 (38.5%)	18 (46.1%)	
Stage III	1	4 (10.3%)	
Stage IV-V	1	3 (7.7%)	
Serum hemoglobin (g/dL)	12.4 ± 2.2	13.4 ± 1.9	0.073
Proteinuria (g/g creatinine)	2.46 ± 0.73	1.83 ± 0.92	0.005

Data are presented as number (% of total) or means ± SD

*BMI* Body mass index, *CKD* Chronic kidney disease, *eGFR* estimated glomerular filtration rate, estimated with Modification of Diet in Renal Disease, *NS* nephrotic syndrome

^a^Group of ‘No progression’ include patients that had spontaneous remission or remission through immunosuppressant or stable status of disease

In the non-sCR group or patients with progression to NS, the most commonly used immunosuppressive regimen was combined corticosteroid and cyclophosphamide (in 22 patients). In addition, oral corticosteroid (4 patients) and corticosteroid combined with cyclosporine (3 patients) were used for treatment. The outcomes of treatments were similar to those of NS patients at baseline [CR in 23 (79.3%) patients, PR in 2 (8.6%) patients]. Primary composite end points developed in 3 (4.6%) patients from the non-sCR group and they also had advanced chronic kidney disease stage with low eGFR at baseline. Therefore, they were treated only with conservative management. The patients who achieved sCR had excellent prognosis and did not show any primary composite outcomes. Although progression to NS occurred in 2 patients in this sCR group, CR was induced with immunosuppressive treatment.

In the logistic regression analyses, serum creatinine and albumin concentrations and the amount of proteinuria at the time of renal biopsy were the significant factors for sCR. Low serum albumin levels at baseline were associated with non-achievement of sCR and high serum albumin levels at baseline were associated with sCR (Odds ratio [OR] = 7.78, *P* = 0.010) (Table 6). Furthermore, low serum albumin concentrations at baseline were associated with progression to NS (OR = 0.015, *P* < 0.001, Table 7).

**Table 6:** Logistic regression analyses for spontaneous complete remission in patients with subnephrotic range proteinuria

	Unadjusted OR	*P*-value	Adjusted OR	*P*-value
Serum creatinine (mg/dl)	0.43 (0.10–1.90)	0.265	0.015 (0.1–0.74)	0.032
Serum albumin (g/dl)	6.10 (2.04–18.26)	0.001	7.78 (1.64–36.89)	0.010
Serum cholesterol (g/dl)	0.99 (0.98–1.00)	0.050	0.99 (0.98–1.00)	0.325
Proteinuria (g/g creatinine)	0.99 (0.98–1.00)	0.006	0.999 (0.998–1.00)	0.038

**Table 7:** Logistic regression analyses for progression to nephrotic syndrome in patients with subnephrotic range proteinuria

	Unadjusted OR	*P*-value	Adjusted OR	*P*-value
Serum creatinine (mg/dl)	0.97 (0.37–2.54)	0.954	0.09 (0.03–2.76)	0.168
Serum albumin (g/dl)	0.31 (0.12–0.79)	0.014	0.015 (0.00–0.15)	<0.001
Serum cholesterol (g/dl)	1.00 (0.99–1.00)	0.050	1.00 (1.00–1.002)	0.314
Proteinuria (g/g creatinine)	1.00 (1.00–1.002)	0.051	1.00 (1.00–1.001)	0.109

## Discussion

In this study, we performed a retrospective investigation to evaluate the effect of response to therapy on the long-term renal function in iMN patients with NS and to determine the prognostic factors in patients with subnephrotic proteinuria. In NS patients, the relapse or persistence of proteinuria was associated with a poor renal survival rate. Furthermore, in the subnephrotic proteinuria patients group, sCR occurred frequently in patients with low proteinuria and serum creatinine levels and with higher serum albumin concentrations at baseline. Furthermore, the serum albumin level at diagnosis was the strongest prognostic factor for progression to NS.

In iMN patients, the conventionally accepted clinical course is that one-third tend to have spontaneous remission, one-third, progressive renal failure, and one-third, stable renal function. In the modern era, the management of iMN has been modified. Anti-proteinuric therapies such as RAAS blockades and strict blood pressure control have been used widely, and there have been significant advances in immunosuppressive therapy [13]. Although definite evidences regarding whether RAAS blockades contribute to remission are lacking [10], the probability of spontaneous remission seems to be high in patients treated with RAAS blockades [14]. Also, the Ponticelli regimen comprising alkylating agents and corticosteroids and the Cattran regimen comprising cyclosporine and low-dose corticosteroid have been found to be beneficial for the induction of remission and reducing the requirement for renal replacement therapy (RRT) [15–17]. Subsequently, many randomised controlled trials have been published. A meta-analysis showed that a combination of alkylating agents and corticosteroids reduced the risk of ESRD and all-cause mortality [18]. Paradoxically, variable courses of iMN and proven benefits of aggressive therapy make it difficult for physicians to choose the appropriate treatment regimen. Although per the KDIGO guidelines, immunosuppressive agents are recommended considering the amount and duration of proteinuria, it is uncertain whether relapse of proteinuria has a negative effect on long-term renal outcomes. Also, management protocols according to the therapeutic response have not yet been established.

Our data showed better prognosis with regard to the remission rate and requirement for RRT than other studies. McQuarrie et al. reported that 76% of the patients achieved at least one PR in 5 years after diagnosis, 32.8% experienced relapse, and 11.9% required RRT [10]. Although the remission rate was similar to that in our study, the probability of relapse in this study (20.8%) was low. The cause of improved outcomes in our study is unclear and might have resulted from the frequent use of RAAS blockades (75.9%) and immunosuppressive therapies (81.9%). Otherwise, it may support the theory that the clinical course in Asian patients is benign as compared to that in the Caucasian population [19–21]. Through analyses of patients presenting with NS, we showed that maintenance of remission state is important for the preservation of long-term renal function. In previous reports, there was a good chance of achieving second remission with treatment even in the relapsed patients, and the prognosis was good compared to the excellent prognosis of persistent remission [10, 22, 23]. Furthermore, because patients showing persistence of proteinuria (defined as no response to treatment in other studies) had poorer renal outcomes than patients who experienced relapse and subsequent remission, it should be emphasised that maintaining remission is important for achieving a favourable long-term outcome. If our results are verified through additional studies, this would affect the clinical decision process regarding the therapeutic regimens. Although the relapse rate was not significantly different for the immunosuppressive agents in this study, it seemed high in the patient group treated with cyclosporine (55.6%) [24]. These possibilities should make physicians pay close attention when choosing immunosuppressive agents, especially for patients with risk factors that affect the long-term renal outcomes.

The sCR was achieved in 26 patients out of 166 patients with NS (15.7%). All the sCR-induced patients have been treated conservatively because of the favorable initial presentation. In NS patients who were treated conservatively without remission induction therapy (30 patients), the parameters reflecting disease severity were advantageous compared to the patients treated with immunosuppressive agents (136 patients). In patients with subnephrotic range proteinuria, the rate of sCR was 35.4% (23 patients out of 65 patients). The initial parameters associated with outcomes were more favorable in patients with subnephrotic proteinuria who did not reach sCR compared to sCR-achieved NS patients. We could explain this discrepancy as a factor of renal function. In patients with subnephrotic proteinuria without sCR, the initial serum creatinine was 1.11 mg/dl. It might reflect an advanced stage of iMN. Therefore, the sCR might not occur in these patients despite of other favorable factors.

In patients with subnephrotic proteinuria, we attempted to create a statistical model that can predict the possibility of spontaneous remission or progression to NS using logistic regression analyses. We found that serum albumin concentration had a high predictive value in forecasting the disease course. Hladunewich et al. reported high proteinuria at baseline in the group that subsequently progressed to NS [11]. In this study, the proteinuria level was also low in patients reaching sCR (1.68 ± 0.88 g/g creatinine vs. 2.36 ± 0.81 g/g creatinine, *P* < 0.003). Furthermore, proteinuria level was one of the prognostic factors for sCR in the multivariate analysis. However, this parameter was not a significant risk factor for progression to NS. This difference suggests that spot urine measurement of proteinuria cannot fully reflect the clinical course of patients because of diurnal variation. Otherwise, the result might have simply been achieved because of a small study size. Serum albumin concentration at the time of diagnosis was revealed to be the strongest prognostic factor for both sCR and the progression to NS among the patients with subnephrotic range proteinuria. We assume that as the disease courses of iMN are shifting, the level of serum albumin reflect this change much faster than other parameters such as a proteinuria amount or serum cholesterol concentration. We ought to measure the serum albumin level serially as well as urine protein in the follow-up of subnephrotic iMN patients.

In patients with subnephrotic range proteinuria depicted in Table 5, the patients who progressed to NS were older and more hypertensive compared to the non-progression group, but those were not significant statistically. However, the level of total cholesterol and the amount of daily proteinuria were high in patients with progression to NS, which were significant statistically. We consider that the concentrations of serum albumin and total cholesterol and the amount of proteinuria are reflecting the severity of iMN. Therefore, their concentrations or amount change parallel in concordance with the disease course. Among the 3 variables, the level of serum albumin was most useful in the risk stratification and this was reported previously [25].

This study has several limitations. This was a retrospective study accomplished with a review of medical records. Therefore, the interpretation might be biased owing to selection error. Because we included records of patients from the year 1989, the treatment regimens were quite different according to the era. Furthermore, there was no standardised regimen for induction and maintenance therapy, and the treatment decisions were totally dependent on the preference of individual physicians. Therefore, these fundamental restrictions could not be avoided in the evaluation of the effect of each treatment.

## Conclusions

We found that the occurrence of relapse or persistence of proteinuria had a negative impact on renal survival in iMN patients with nephrotic syndrome. In addition, low serum albumin levels at baseline were associated with non-achievement of sCR and progression to NS.

## Abbreviations

CR: Complete remission
eGFR: Estimated glomerular filtration rat
ESRD: End stage renal disease
HR: Hazard ratio
iMN: Idiopathic membranous nephropathy
NS: Nephrotic syndrome
OR: Odds ratio
PR: Partial remission
RAAS: Renin-angiotensin-aldosterone system
RRT: Renal replacement therapy
sCR: Spontaneous complete remission
uPCR: Urine protein creatinine ratio
